# Molecular mechanisms leading to ceftolozane/tazobactam resistance in clinical isolates of *Pseudomonas aeruginosa* from five Latin American countries

**DOI:** 10.3389/fmicb.2022.1035609

**Published:** 2022-10-24

**Authors:** María F. Mojica, Elsa De La Cadena, Rafael Ríos, Juan Carlos García-Betancur, Lorena Díaz, Jinnethe Reyes, Cristhian Hernández-Gómez, Marcela Radice, Ana C. Gales, Paulo Castañeda Méndez, José M. Munita, Christian José Pallares, José R. W. Martínez, María Virginia Villegas

**Affiliations:** ^1^Grupo de Investigación en Resistencia Antimicrobiana y Epidemiologia Hospitalaria, Universidad El Bosque, Bogotá, Colombia; ^2^Department of Molecular Biology and Microbiology, School of Medicine, Case Western Reserve University, Cleveland, OH, United States; ^3^Cleveland VA Medical Center for Antimicrobial Resistance and Epidemiology (Case VA CARES), Case Western Reserve University, Cleveland, OH, United States; ^4^Research Service, VA Northeast Ohio Healthcare System, Cleveland, OH, United States; ^5^Molecular Genetics and Antimicrobial Resistance Unit, Universidad El Bosque, Bogotá, Colombia; ^6^Millenium Initiative for Collaborative Research on Bacterial Resistance (MICROB-R), Santiago, Chile; ^7^Universidad de Buenos Aires, Facultad de Farmacia y Bioquímica, Buenos Aires, Argentina; ^8^Consejo Nacional de Investigaciones Científicas y Técnicas (CONICET), Buenos Aires, Argentina; ^9^Universidade Federal de São Paulo, Division of Infectious Diseases, Brazil; ^10^Hospital Médica Sur, Ciudad de México, Mexico; ^11^Genomics and Resistant Microbes (GeRM) Instituto de Ciencias e Innovación en Medicina, Facultad de Medicina, Clínica Alemana, Universidad del Desarrollo, Santiago, Chile; ^12^Clínica Imbanaco, Grupo Quiron, Cali, Colombia

**Keywords:** ceftolozane/tazobactam, *Pseudomonas aeruginosa*, antibiotic resistance, molecular mechanisms, Latin America

## Abstract

**Objectives:**

Identify molecular mechanisms responsible for the *in vitro* non-susceptibility to ceftolozane/tazobactam (TOL) in a group of 158 clinical isolates of *Pseudomonas aeruginosa* from five Latin American countries collected before the introduction of TOL into the clinical practice.

**Methods:**

Clinical isolates of *P. aeruginosa* (*n* = 504) were collected between January 2016 and October 2017 from 20 hospitals located in Argentina, Brazil, Chile, Colombia, and Mexico. Minimum inhibitory concentrations (MICs) to TOL were determined by standard broth microdilution and interpreted according to CLSI breakpoints. Initially, production of carbapenemases in TOL non-susceptible isolates was assessed by Rapidec® followed by qPCR to detect *bla*_KPC_, *bla*_NDM-1_, *bla*_VIM_, and *bla*_IMP_. Illumina® WGS was performed for isolates in which non-susceptibility to TOL was not mediated by carbapenemases.

**Results:**

A total of 158 (31.3%) isolates were non-susceptible to TOL. In 74 (46.8%) of these isolates, non-susceptibility to TOL was explained by the production of at least one carbapenemase. WGS revealed that some isolates carried ESBLs, mutated *bla*_PDC_ and *ampD*, associated with decreased susceptibility to TOL.

**Conclusion:**

Substitutions found in PDC and carbapenemase production were the most common presumed mechanisms of resistance to TOL detected in this study. This study shows that epidemiological surveillance is warranted to monitor the emergence of novel mechanisms of resistance to TOL that might compromise its clinical utility.

## Introduction

Increased bacterial resistance to antibiotics in conjunction with the lack of new drugs has become a major public health concern worldwide. This is especially worrisome in the case of *Pseudomonas aeruginosa,* a non-fermenter Gram-negative bacillus recognized by its ability to become resistant to multiple drugs, by simultaneously expressing multiple enzymatic and non-enzymatic mechanisms of resistance (Pang et al., 2019). The lack of therapeutic alternatives means that infections caused by these multidrug resistant (MDR) bacteria pose a considerable threat regarding morbidity and mortality worldwide (Mesaros et al., 2007).

Ceftolozane/tazobactam (TOL) is a combination of a fifth-generation cephalosporin with a well-known β-lactamase inhibitor (Liscio et al., 2015). Ceftolozane exhibits a high affinity for penicillin binding proteins PBP1b, PBP1c, and PBP3, improved outer membrane permeability, increased stability against efflux and enhanced stability against chromosomal AmpC β-lactamase, resulting in potent *in vitro* activity against *P. aeruginosa* (Bush and Bradford, 2016). However, due to the inability of tazobactam to inhibit carbapenemases, TOL is not active against carbapenemase producers (Yahav et al., 2021). Besides this intrinsic liability, other resistance mechanisms to TOL have been described. Among these, mutational derepression of *ampC*, the gene encoding the inducible Pseudomonas-derived cephalosporinase (PDC) β-lactamase, is one of the most frequently reported. Genes involved in *ampC* overexpression include *amp*R, *amp*G, *amp*D, and *dac*B (Cabot et al., 2014). Substitutions in the regulator AmpR (e.g., D135N/G) have been associated with the development of high-level resistance to TOL (Fournier et al., 2021). Resistance to TOL has also been described in *P. aeruginosa* strains producing PDC with improved catalytic activity toward ceftolozane, due to substitutions at the Ω-loop (F147L, Q157R, G183D, V211A, G214R, E219K, E219G, Y221H, E247K, or V356I; Cabot et al., 2014). Ceftolozane activity does not seem to be significantly impaired by active efflux mechanisms such as the Mex pumps (Takeda et al., 2007).

Herein, we describe the molecular mechanisms of resistance to TOL present in clinical isolates of *P. aeruginosa* recovered in 20 different hospitals in 5 Latin American countries prior to the introduction of this antibiotic combination into the clinical practice.

## Materials and methods

### Bacterial isolates and susceptibility testing

This study was conducted on 504 clinical isolates of *P. aeruginosa* with the so called multidrug resistant (MDR) phenotype. Isolates were collected from 20 medical institutions located in Colombia, Brazil, Argentina, Chile, and Mexico from January 2016 to October 2017, prior to the introduction of TOL into the clinical practice in these countries. MDR was defined by non-susceptibility to ≥1 agent in ≥3 classes that are typically active against *P. aeruginosa* (Magiorakos et al., 2012).

Bacterial identification was performed using the MALDI-TOF MS system (BioMérieux, France). Minimum inhibitory concentrations (MICs) for TOL were determined by broth microdilution, following the guidelines of the Clinical and Laboratory Standard Institute (CLSI) M100 (CLSI, 2021). *Escherichia coli* ATCC 25922 and *P. aeruginosa* ATCC 27853 were used as controls.

### Characterization of resistance mechanisms

Presence of carbapenemases in TOL non-susceptible *P. aeruginosa* isolates (MIC ≥ 8/4) was determined by Rapidec® Carba-NP test (BioMérieux, France), following manufacturer’s protocol and, molecular detection of carbapenemases was performed on all Carba-NP-positive isolates by *in-house* qPCR assays targeting genes encoding the following groups of carbapenemases: KPC, NDM, VIM, and IMP (Correa et al., 2015). *P. aeruginosa* isolates that were non-susceptible to TOL and did not harbor any of these carbapenemases, were randomly selected to undergo whole genome sequencing (WGS).

### WGS analysis

Genomic DNA was extracted using DNeasy Blood and Tissue Kit (Qiagen, Hilden, Germany). DNA quality was verified by agarose gel electrophoresis and quantified using Qubit 2.0 fluorometer (Invitrogen, Life Technologies Italia, Monza, Italy). Paired-end libraries were prepared from 1 ng of total bacterial DNA using Nextera XT DNA Sample Preparation kit and Nextera XT Index kit (Illumina Inc., San Diego, CA). Library concentration and average fragment size were calculated by Qubit 2.0 fluorometer and Agilent 2100 bioanalyzer (Agilent technologies, Santa Clara, CA), respectively. Sequences were obtained on the Illumina MiSeq platform with 300 nt paired end reads to achieve a coverage of about 80X per base, using MiSeq V3 flow cell. Raw reads underwent a series of steps for quality filtering which included a general quality check and the trimming of low-quality ends. *de novo* assembly were performed using Spades v3.13 software (Bankevich et al., 2012). Annotation was done by using RAST server, and the presence of resistance determinants were determined by BLAST tool and ResFinder database (Zankari et al., 2012). The genome of *P. aeruginosa* PAO1 (GenBank ID: NC_002516.2) was used as reference to look for known substitutions associated with TOL resistance in AmpC and its regulators, AmpR, AmpG, AmpR, AmpD, and DacB. MLST 1.8 server^1^ was used to perform MLST of *P. aeruginosa* isolates, based on the seven housekeeping genes (*acsA*, *aroE, guaA*, *mutL*, *nuoD*, *ppsA*, and *trpE*), as previously described (Larsen et al., 2012). The pangenome of the isolates was determined with Roary v3.13.0 (Seemann, 2014). A maximum likelihood (ML) phylogenetic tree using a core genome definition of 99% was performed with RAxML 8.2.12 (Stamatakis, 2014) with 400 bootstrap iterations using a general time reversible (GTR) substitution model with four gamma rate categories. Recombination events were assessed with Clonal Frame ML v1.12, using the core genome alignment and the ML tree (Didelot and Wilson, 2015). All phylogenomic trees were visualized with the interactive Tree Of Life (iTOL) v6 tool (Letunic and Bork, 2021).

## Results

Among 504 *P. aeruginosa* isolates recovered in 5 Latin American countries, 228 (45.2%) were not susceptible to piperacillin-tazobactam, 295 (58.5%) were not susceptible to ceftazidime, 232 (46%) were non-susceptible to cefepime, and 158 (31.3%) were non-susceptible to TOL. The complete susceptibility profile of these isolates was previously published (García-Betancur et al., 2022). From the TOL non-susceptible isolates, the Rapidec® Carba-NP detected the production of a carbapenemase in 74 (46.8%) isolates. Results from the carbapenemase identification by qPCR performed on these 74 strains are shown in Table 1. TOL non-susceptibility in all these isolates was explained by the presence of at least one carbapenemase: KPC (*n =* 31), VIM (*n =* 32), IMP (*n =* 3), KPC + VIM (*n =* 7), and SPM (*n =* 1). From the remaining 84 isolates, 57 (67.8%) were randomly chosen to be sequenced.

**Table 1:** Phenotypic and molecular screening of carbapenemases in 74 TOL non-susceptible clinical isolates of *Pseudomonas aeruginosa* from 5 Latin American countries.

Country	Number of isolates	TOL NS (%)	Carba-NP (+)	PCR (+)				
				*bla*_KPC_	*bla*_VIM_	*bla*_KPC_ *+ bla*_VIM_	*bla*_IMP_	*bla*_SPM_
Argentina	30	9 (30%)	2	0	0	0	2	0
Brazil	40	12 (30%)	2	1	0	0	0	1
Chile	62	12 (19,3)	7	3	4	0	0	0
Colombia	246	82 (33,3%)	58	27	24	7	0	0
Mexico	126	43 (34,1%)	5	0	4	0	1	0

**Figure 1:**
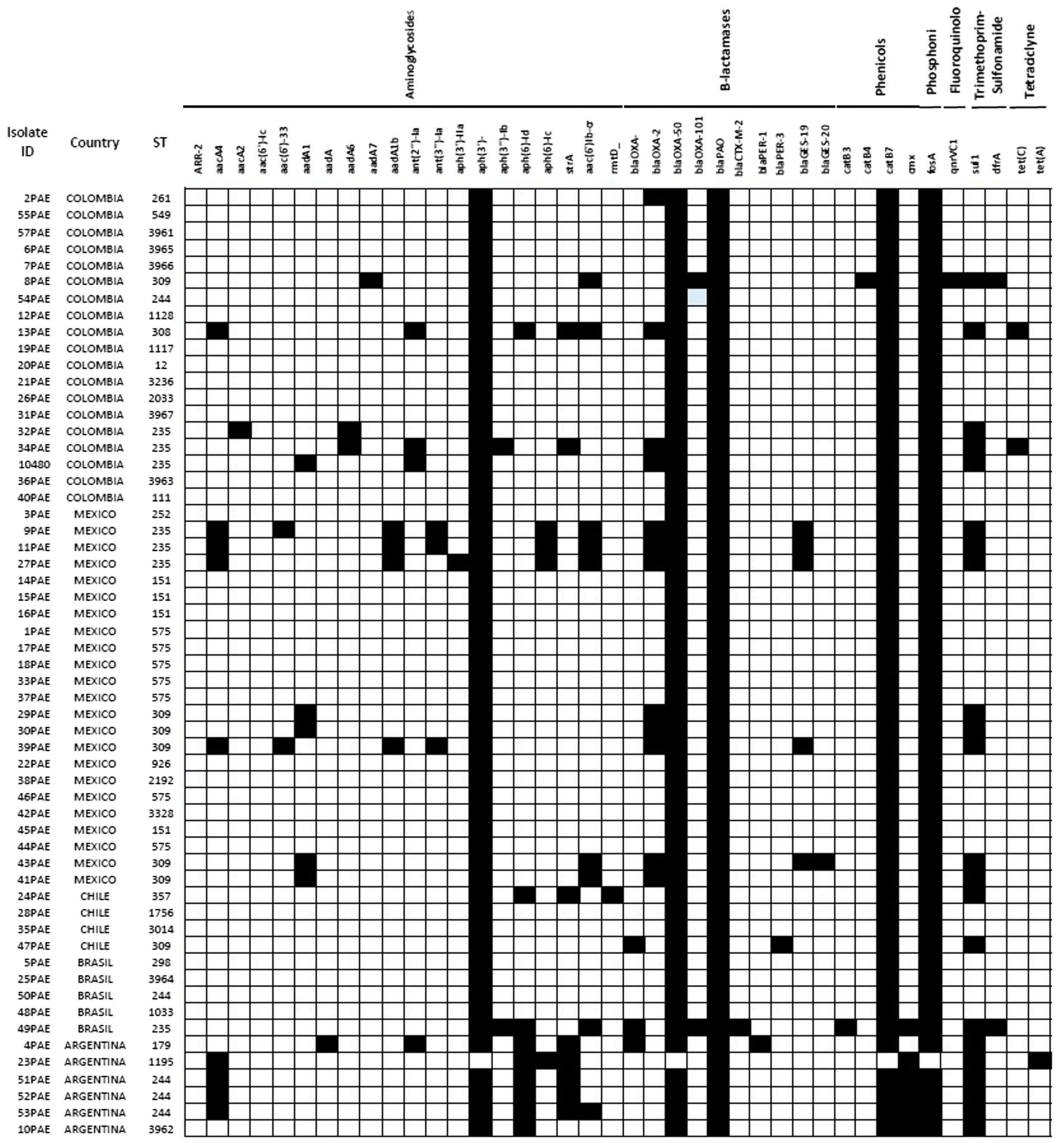
Principal phenotypic and genomic characteristics of the *Pseudomonas aeruginosa* TOL-resistant isolates sequenced. Black squares mean presence, white squares mean absence.

A summary of the resistome encoded by the 57 sequenced isolates is presented in Supplementary File 1. As expected, in all isolates the genes of the chromosomally-encoded β-lactamases PDC and OXA-50/PoxB were found; among the acquired β-lactamases genes *bla*_OXA-2_, *bla*_OXA-4_, *bla*_OXA-101_, *bla*_PER-1_, *bla*_PER-1,_ *bla*_GES-19,_ and *bla*_GES-20_ were detected in 12, 6, 1, 1, 1, 5, and 1 isolates, respectively. One isolate harbored both *bla*_OXA-4_ and *bla*_PER-1_, whereas five isolates carried *bla*_OXA-2_ and *bla*_GES-19_ genes. Interestingly, one isolate harbored *bla*_GES-19_ and *bla*_GES-20_ in tandem. Analysis of the inferred amino acid sequence revealed that the most common PDC was PDC-3 (*n* = 18) followed by PDC-1 (*n* = 9), PDC-35 (*n* = 7), PDC-37 (*n* = 6), PDC-19a (*n* = 4), PDC-5 (*n* = 3), PDC-8 and PDC-16 (*n* = 2 each). PDC-11, PDC-24, PDC-33, PDC-64, PDC-67, and PDC-6 were each produced by one isolate (Figure 1).

**Figure 2:**
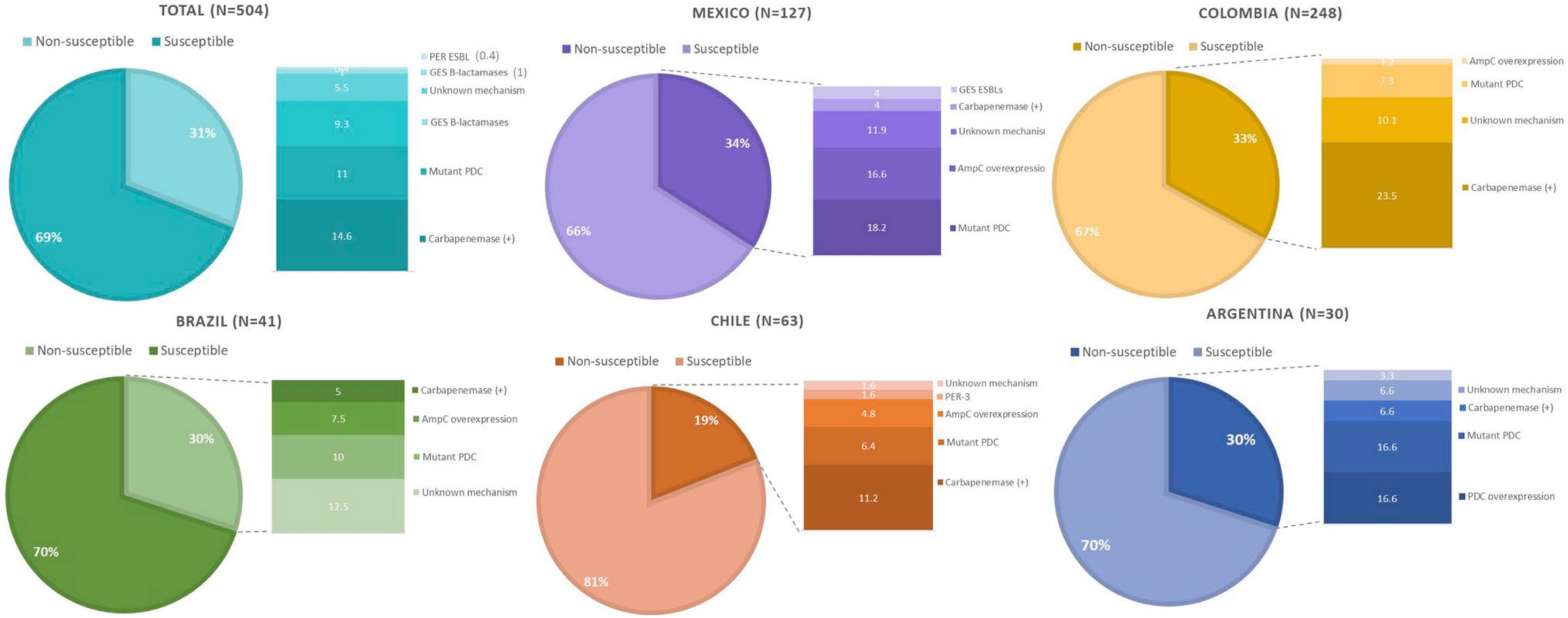
Overview of the molecular mechanisms leading to TOL resistance in clinical isolates of *Pseudomonas aeruginosa* collected in five Latin American countries.

Substitutions in PDC previously associated with decreased susceptibility to TOL, namely G1D, T79A, V179L, V329I, and G364A, were found in 20, 54, 22, 13, and 23 isolates, respectively. Twelve isolates were found to harbor all five mutations (G1D, T79A, V178L, V329I, and G364A), and 8 harbored four of these changes (G1D, T79A, V179L, and G364A). Only one isolate had mutation in V211A accompanied by T79A and G364A. On the other hand, AmpR substitutions E114A, G283E, and M288R were detected in 6, 20, and 21 isolates, respectively. Only four isolates carried all three mutations. Lastly, the mechanisms of resistance to TOL of isolate 40PAE could not be inferred, as mutations in the genes associated to TOL resistance (*ampC*, *ampR*, *ampD*, *ampG*, PBP3, and PBP4) were not identified. An overall summary of the results per country is provided in Figure 2.

**Figure 3:**
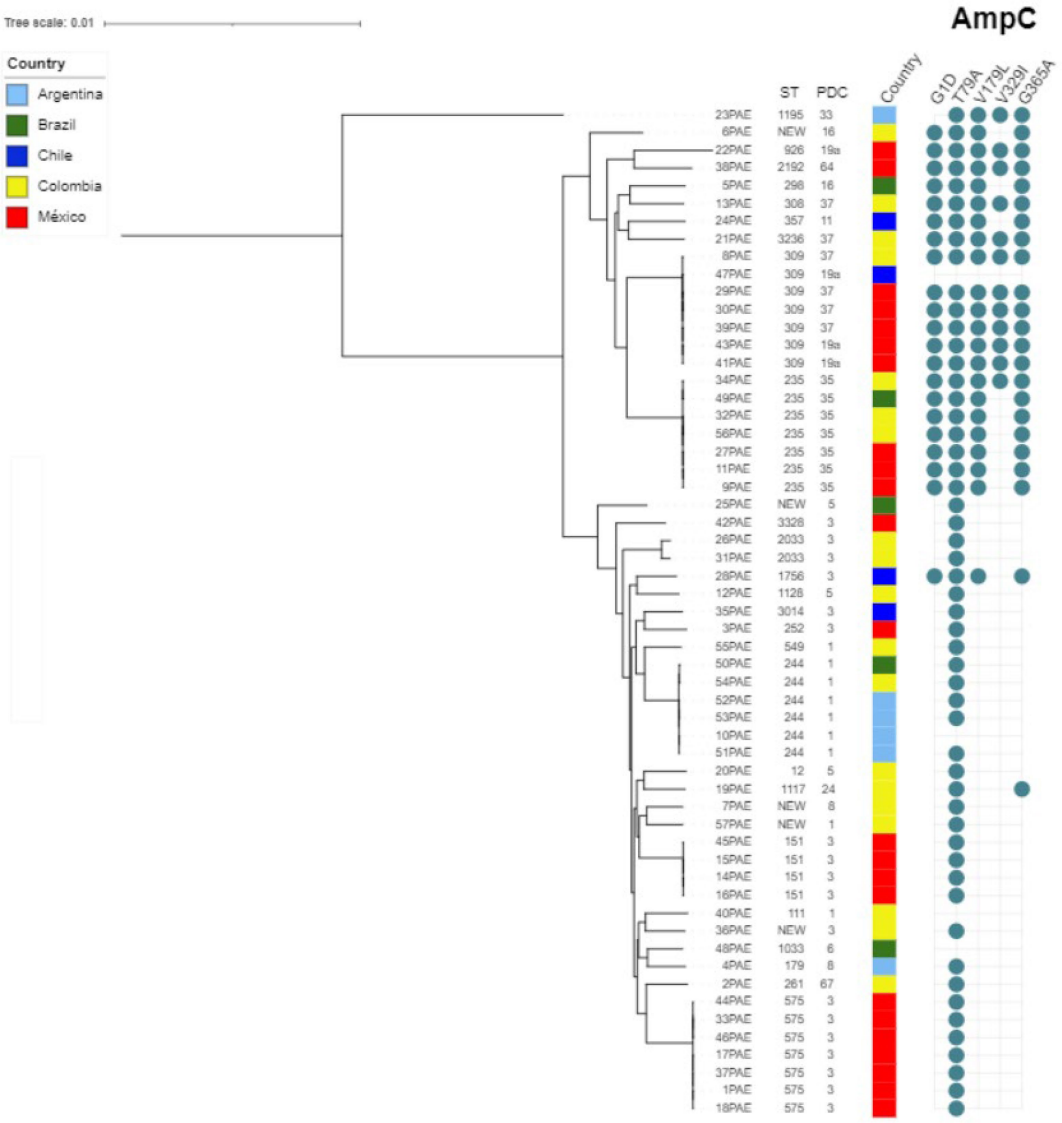
Genetic relatedness among TOL non-susceptible *Pseudomonas aeruginosa* isolates. The phylogenetic tree was obtained by RAxML from the core genome (ortholog genes present in more than 95% of included genomes) and drawn using the iTOL tool (Letunic and Bork, 2007).

As for the molecular epidemiology of the sequenced strains, *in silico* multilocus sequence typing (MLST) revealed the circulation of 32 different sequence types (STs), including the high-risk clones ST235 and ST111, alongside several new STs. However, a predominant ST or clonal complex was not identified, as can be seen in Figure 3.

## Discussion

*Pseudomonas aeruginosa* is a leading nosocomial pathogen with a remarkable ability to develop resistance. Consequently, the emergence of strains displaying the MDR phenotype is a serious public health threat that affects patients in specialized units such as intensive care units, hematology-oncology wards or burn units (Magiorakos et al., 2012; García-Betancur et al., 2021).

Ceftolozane is a novel expanded-spectrum cephalosporin, developed with the intention of being a powerful antipseudomonal β-lactam antibiotic. Structurally, ceftolozane is closely related to ceftazidime, which is a “first-line” treatment of *P. aeruginosa* infections, and also targets primarily PBP3 (Moyá et al., 2012). Its innovative design is intended to make the molecule stable to the hydrolysis by PDC, the chromosomally encoded class C β-lactamase of *P. aeruginosa* (Takeda et al., 2007). However, as with other oxyimino-cephalosporins, ceftolozane is labile to the hydrolysis by some class A extended-spectrum β-lactamases (ESBLs) such as PER and GES, but not CTX-M-type enzymes; class D ESBLs (e.g., OXA-2 and OXA-10-like enzymes); and class A and B carbapenemases like KPC-, VIM-, IMP-, and NDM-like enzymes, all of which have been described in *P. aeruginosa* (Rodríguez-Martínez et al., 2009; Livermore et al., 2010; Ortiz De La Rosa et al., 2019). Tazobactam is a penicillin-based sulfone derivative β-lactamase inhibitor that inactivates most class A β-lactamases (e.g., CTX-M), with variable activity against class C and class D β-lactamases (Aronoff et al., 1984; Bush and Jacoby, 2010). Tazobactam do not inhibit any carbapenemase (Drawz and Bonomo, 2010).

Given this profile, TOL is not expected to be active against *P. aeruginosa* isolates that carry classes A or B carbapenemases, or classes A, C, and D ESBLs that are not readily inhibited by tazobactam (e.g., GES-6, PER-1, FOX-4, and OXA-539). Accordingly, 74 (46.8%) of our TOL resistant isolates were positive for the presence of at least one carbapenemase, mainly KPC and VIM. Of note, seven (9.5%) isolates from Colombia carried simultaneously KPC and VIM carbapenemases. Our group identified *P. aeruginosa* isolates co-harboring KPC and VIM for the first time in 2012, and according to local surveillance studies, strains co-producing these two carbapenemases and belonging to different STs have continuing spreading throughout the country (Villegas et al., 2007; Vanegas et al., 2014; Pacheco et al., 2019; Rada et al., 2021).

Resistance to TOL in isolates that do not carry carbapenemases is frequently mediated by amino acid substitutions, insertions, and/or deletions in PDC. To date, over 300 PDC variants have been reported.^2^ Several PDC variants such as PDC-50 (V213A); PDC-74, PDC-75, and PDC-78 (G216R); PDC-79 and PDC-86 (E221K); PDC-80 (E221G); and PDC-85 (Y223H) originally identified in highly MDR *P. aeruginosa* clinical isolates conferred resistance to several antibiotics, including TOL (Berrazeg et al., 2015). However, none of those PDC variants were found in our study. Some other PDC substitutions previously reported associated with TOL non-susceptibility G1D, T79A, V179L, V329I, and G364A were found in 20, 54, 22, 13, and 23 isolates, respectively. Twelve isolates were found to harbor all five mutations, and 8 isolates harbor four mutations (G1D, T79A, V179L, and G364A). High-level resistance to TOL has been reported in isolates overexpressing mutated *bla*_PDC_ presenting multiple substitutions that result in structural modifications in the enzyme (Cabot et al., 2014).

Another successful way to attain TOL resistance is through overexpression of *bla*_PDC_. De-repression of *bla*_PDC_ can result from mutations in the genes involved in its regulation, namely *ampR, ampG,* and *ampD. P. aeruginosa* isolates overproducing PDC have been shown to have elevated TOL MICs (Papp-Wallace et al., 2020). The G154R and D135N/G substitutions in AmpR have been shown to drive PDC overexpression, leading to TOL resistance (Cabot et al., 2014). We did not find these mutations in our isolates. Instead, AmpR substitutions E114A, G283E, and M288R were detected in 6, 20, and 21 isolates, respectively. Only four isolates carried all three mutations. Further studies are needed to determine the effect of these substitutions, individually and collectively, on the levels of PDC production and TOL resistance. Alternatively, mutational inactivation of *amp*D genes have been commonly documented to lead to *bla*_PDC_ hyperexpression (Papp-Wallace et al., 2020). One isolate showed frameshifts in the *ampD* gene and eight had premature stop codon in H157. The H157R substitution in AmpD has been previously reported in *P. aeruginosa* isolates resistant to TOL and ceftazidime-avibactam (Juan et al., 2006; Zamudio et al., 2019). *In vitro* studies have shown that specific mutations in PBP3 (encoded by *ftsI*) and PBP4 (encoded by *dac*B) play a role in the emergence of β-lactam resistance. In specific, TOL resistance has been associated with the R504C/R504H and F533L substitutions in PBP3. However, we did not find any of these substitutions in our isolate. Instead, 11 isolates had a N117S substitution, which has not been reported associated with TOL resistance. Further studies are required to determine the consequence of that PBP3 substitution in ß-lactams binding. Finally, none of our isolates carried any mutated PBP-2 or PBP-4.

ESBLs have also been associated with decreased susceptibility to TOL. Indeed, ESBLs such as GES-like, PER-1, BEL-1, BEL-2, and VEB-1, and some OXA enzymes like OXA-2 variants, OXA-10, and OXA-14 can significantly affect the efficacy of TOL (Vanegas et al., 2014; Rada et al., 2021). In this study only two isolates, one from Argentina and one from Chile were found to carry PER-1 and PER-3, respectively. In the case of the Argentinian strain (isolate 4PAE), it also harbored a mutated *bla*_PDC_ that leads to the T79A substitution, previously associated with decreased susceptibility to TOL. Of notice, we identified five strains carrying *bla*_GES_, all of them from Mexico. Genes encoding ESBL GES-19 and carbapenemase GES-20 are of high prevalence among imipenem resistant *P. aeruginosa* in that country (Garza-Ramos et al., 2015). As mentioned before, we found one isolate harboring *bla*_GES-19_ and *bla*_GES-20_ displaying high-level resistance to TOL (isolate 43PAE; MIC > 128 mg/L). *P. aeruginosa* strains belonging to the ST309 producing GES-19 and GES-26 have been reported displaying resistance to TOL in Mexico (Khan et al., 2019). In this study, we found one strain from Mexico belonging to the ST309 that although only carried *bla*_GES-19_, also displayed high-level resistance to TOL (isolate 39PAE; MIC > 128 mg/L). Lastly, we also found three isolates belonging to the high-risk clone ST235 producing GES-19 and OXA-2 in the samples from Mexico.

Our study has many limitations. Due to budget restrictions, we could only sequence a fraction of the isolates displaying resistance to TOL that did not produce a carbapenemase. That impaired us to have the complete picture of the molecular epidemiology of all the clinical isolates of *P. aeruginosa* and may explain why we only found a handful of isolates belonging to the high-risks clones ST111, ST175 and ST235. Also, we could only presume overexpression of *bla*_PDC_ based on the mutation in the regulator genes. qRT-PCR and westerns blots are necessary to demonstrate hyperexpression of that gene. Finally, we are reporting known mechanisms of reduced susceptibility to TOL in *P. aeruginosa*. More studies are needed to investigate novel mechanisms of resistance to TOL and to other novel antibiotic combinations in this important pathogen. Despite the study limitations, we believe it adds to the knowledge of circulating mechanisms of resistance in *P. aeruginosa* in Latin America and emphasizes the genomic plasticity of *P. aeruginosa* that allows it to develop resistance to antibiotics even before its clinical use.

In conclusion, our results show that production of carbapenemases and amino acid substitution in the chromosomally encoded PDC, leading to structural modifications and increased hydrolytic activity to TOL of the cephalosporinase, were the main mechanism leading to TOL resistance in selected MDR *P. aeruginosa* isolates in Latin America. Surveillance studies with contemporary isolates are needed to identify current mechanisms of resistance to this drug due to widespread use in the clinical practice.

## Data availability statement

The datasets presented in this study can be found in online repositories. The names of the repository/repositories and accession number(s) can be found at: https://www.ncbi.nlm.nih.gov/, PRJNA729968.

## Author contributions

MV, EC, MM, and CH-G conceptualized and designed the study. MV, EC, MM, and JG-B performed the validation and formal analysis of the study. MR, AG, PC, JMu, and CP performed the formal analysis, reviewed and edited the manuscript. RR, LD, JR, and JMa performed the bioinformatic analysis and reviewed the manuscript. MM, EC, JG-B, and MV: writing, original draft preparation, and review and editing. All authors contributed to the article and approved the submitted version.

## Funding

This study was supported by Merck & Co. Inc., under the grant ref. 10020230. The funder was not involved in the study design, collection, analysis, interpretation of data, the writing of this article or the decision to submit it for publication.

## Conflict of interest

CH-G is currently employed by MSD. JMu received funding from FONDECYT #1211947 from the Agencia Nacional de Investigation y Desarrollo (ANID), Government of Chile. MV have received consulting fees from MSD, Pfizer; WEST, and BioMérieux, none of which had any involvement in this study. 

The remaining authors declare that the research was conducted in the absence of any commercial or financial relationships that could be construed as a potential conflict of interest.

## Publisher’s note

All claims expressed in this article are solely those of the authors and do not necessarily represent those of their affiliated organizations, or those of the publisher, the editors and the reviewers. Any product that may be evaluated in this article, or claim that may be made by its manufacturer, is not guaranteed or endorsed by the publisher.

## Footnotes

^1^ https://cge.cbs.dtu.dk/services/pMLST/

^2^ https://arpbigidisba.com
